# A Simple Analytical Model for Magnetization and Coercivity of Hard/Soft Nanocomposite Magnets

**DOI:** 10.1038/s41598-017-04632-6

**Published:** 2017-07-10

**Authors:** Jihoon Park, Yang-Ki Hong, Woncheol Lee, Seong-Gon Kim, Chuangbing Rong, Narayan Poudyal, J. Ping Liu, Chul-Jin Choi

**Affiliations:** 1 Department of Electrical and Computer Engineering and MINT Center, The University of Alabama Tuscaloosa, Alabama, 35487 USA; 2 Powder and Ceramics Division, Korea Institute of Materials Science, Changwon, 51508 Republic of Korea; 3 Department of Physics & Astronomy and Center for Computational Sciences, Mississippi State University, Mississippi State, Mississippi 39762 USA; 4 Department of Physics, University of Texas at Arlington, Arlington, Texas 76019 USA

## Abstract

We present a simple analytical model to estimate the magnetization (*σ*_s_) and intrinsic coercivity (*H*_ci_) of a hard/soft nanocomposite magnet using the mass fraction. Previously proposed models are based on the volume fraction of the hard phase of the composite. However, it is difficult to measure the volume of the hard or soft phase material of a composite. We synthesized Sm_2_Co_7_/Fe-Co, MnAl/Fe-Co, MnBi/Fe-Co, and BaFe_12_O_19_/Fe-Co composites for characterization of their *σ*_s_ and *H*_ci_. The experimental results are in good agreement with the present model. Therefore, this analytical model can be extended to predict the maximum energy product (*BH*)_max_ of hard/soft composite.

## Introduction

There are two issues in rare-earth (RE) permanent magnets (PM) for full applications. One is RE mineral security, and the other is a low Curie temperature of Nd-Fe-B magnet. The figure of merit of PM is its maximum energy product, (*BH*)_max_. The (*BH*)_max_ can be estimated as (*BH*)_max_ = (*B*_r_)^2^/4 for *H*_ci_ > *B*_r_/2 or (*BH*)_max_ = (*B*_r_ − *H*_ci_)*H*_ci_ for *H*_ci_ < *B*_r_/2 ^1^. *B*_r_ is the remanent magnetic flux density, and *H*_ci_ is the intrinsic coercivity, which is mainly controlled by the magnetocrystalline anisotropy constant (*K*). Therefore, high *B*_r_ and *H*_ci_ are needed for a large (*BH*)_max_. In addition, the PM must also have a corresponding high Curie temperature (*T*_c_) to retain the figure of merit at typical operating temperatures. In an effort to increase the (*BH*)_max_ of RE-free permanent magnets, concepts of exchange coupling between hard and soft magnetic phases have been proposed^2, 3^. Exchange coupling makes full use of high *H*_ci_ from the hard phase and *B*_r_ from the soft phase of a hard/soft composite magnet. Therefore, a large (*BH*)_max_ of a composite magnet can be achieved. In the magnetic exchange coupled composite, the magnetization direction of the soft phase is pinned to the magnetization direction of the hard phase^4^. This implies that the exchange coupled two-phase magnet behaves like a single-phase magnet. However, the soft magnetic phase needs to be thinner than twice the domain wall thickness (2*δ*_w_) of hard magnetic phase for full exchange coupling^2^. Thus, the increasing rate of (*BH*)_max_ with the amount of soft phase is limited. Although the previously proposed models^2, 3^ predict the magnetization in the unit of emu/cm^3^ (*M*) and *K* of an exchange coupled thin film magnet reasonably well, a model directly applicable to a powdered (bulk) hard/soft nanocomposite magnets is not yet reported. In this paper, we developed a model for the magnetization in the unit of emu/g (*σ*_s_) and *H*_ci_ of powdered hard/soft composite based on, experimentally accessible, the mass fraction of hard and soft magnetic phases instead the volume fraction. The prediction of the developed model was compared with the experimental *σ*_s_ and *H*_ci_ of Sm_2_Co_7_/Fe-Co, MnAl/Fe-Co, MnBi/Fe-Co, and BaFe_12_O_19_(BaM)/Fe-Co, where Sm_2_Co_7_, MnAl, MnBi, and BaM are hard magnetic phases, and Fe-Co is a soft magnetic phase.

## Derivation of Equations

We now derive the equations for *σ*_s_ and *H*_ci_ in terms of mass fraction of composite. According to theoretical studies on a two-phase composite magnet, the saturation magnetization^2^ and anisotropy constant^3^ of a composite can be expressed as:

1 $$M={f}_{s}{M}_{s}+{f}_{h}{M}_{h}$$

and

2 $$K={f}_{s}{K}_{s}+{f}_{h}{K}_{h},$$

where *M* is the saturation magnetization, *K* is the magnetocrystalline anisotropy constant, and *f* is the volume fraction. *h* and *s* in the subscript denote hard and soft phases, respectively. Because of the experimental difficulty of obtaining *M* and *K* (per unit volume) of powdered composite, we seek to develop expressions for *σ*_s_ and *H*_ci_ (per unit mass) of a two-phase magnetic composite using experimentally accessible *σ*_s_ and *H*_ci_ for both hard and soft phases. Noting that *M* in Eq. (1) is magnetic moment per unit volume (typically in the unit of emu/cm^3^), they can be expressed as:

$$M=\frac{{\rm{magneticmoment}}}{{\rm{volume}}}=\frac{{\rm{magneticmoment}}}{{\rm{mass}}}\cdot \frac{{\rm{mass}}}{{\rm{volume}}}=\sigma \cdot \rho $$

where $\sigma$ is the saturation magnetization (per unit mass in the unit of emu/g) and *ρ* is the mass density (in the unit of g/cm^3^). Therefore, the *σ* (the subscript *s* will be omitted for now to avoid the confusion with quantities for soft phase) of two-phase magnetic composites can be written as:

3 $$\sigma =\frac{{\sigma }_{h}{\rho }_{h}\,{f}_{h}+{\sigma }_{s}{\rho }_{s}\,{f}_{s}}{{\rho }_{h}\,{f}_{h}+{\rho }_{s}\,{f}_{s}}.$$

*H*_ci_ due to magnetocrystalline anisotropy^5^ is

4 $${H}_{ci}=\frac{\alpha K}{\sigma \rho },$$

where α is a constant dependent on the crystal structure and degree of alignment. *α* is 2 in the case of aligned particles^6^ while for unaligned (random) particles, *α* can have different values for different crystals (for instance, 0.64 for cubic crystals^7^ and 0.96 for uniaxial crystals). Then, *H*_ci_ of the two-phase magnetic composite can be modified to equation (5) by combining Eqs (2) and (4):

5 $${H}_{ci}=\alpha \frac{(1-{f}_{h}){K}_{s}+{f}_{h}{K}_{h}}{(1-{f}_{h}){\sigma }_{s}{\rho }_{s}+{f}_{h}{\sigma }_{h}{\rho }_{h}}.$$

By replacing *K* in Eq. (5) using Eq. (4), Eq. (5) becomes

6 $${H}_{ci}=\frac{{\sigma }_{h}{\rho }_{h}{H}_{h}\,{f}_{h}+{\sigma }_{s}{\rho }_{s}{H}_{s}\,{f}_{s}}{{\sigma }_{h}{\rho }_{h}\,{f}_{h}+{\sigma }_{s}{\rho }_{s}\,{f}_{s}},$$

where *H*_*h*_ and *H*_*s*_ are the intrinsic coercivities of hard and soft phases, respectively. Therefore, the *H*_*ci*_ of a composite can now be estimated by experimental *H*_*h*_ and *H*_*s*_ instead of the *K*_*h*_ and *K*_*s*_. Furthermore, since it is difficult to measure the volume fraction of a powdered sample, we further develop equations for *σ*_s_ and *H*_ci_ of a two-phase composite in terms of the mass fraction. Since *f*_h_ is the volume fraction of hard magnetic phase, i.e.,

7 $${f}_{h}=\frac{{V}_{h}}{{V}_{h}+{V}_{s}},$$

where *V* is the volume. Therefore, Eqs (3) and (6) become

8 $$\sigma =\frac{{\sigma }_{h}{V}_{h}{\rho }_{h}+{\sigma }_{s}{V}_{s}{\rho }_{s}}{{V}_{h}{\rho }_{h}+{V}_{s}{\rho }_{s}}$$

and

9 $${H}_{ci}=\frac{{\sigma }_{h}{H}_{h}{V}_{h}{\rho }_{h}+{\sigma }_{s}{H}_{s}{V}_{s}{\rho }_{s}}{{\sigma }_{h}{V}_{h}{\rho }_{h}+{\sigma }_{s}{V}_{s}{\rho }_{s}},$$

respectively. Dividing both the numerator and denominator in Eqs (8) and (9) by the total mass, i.e. (*V*_h_ *ρ*_h_ + *V*_s_ *ρ*_s_), we get:

10 $$\sigma =\frac{{\sigma }_{h}\frac{{V}_{h}{\rho }_{h}}{{V}_{h}{\rho }_{h}+{V}_{s}{\rho }_{s}}+{\sigma }_{s}\frac{{V}_{s}{\rho }_{s}}{{V}_{h}{\rho }_{h}+{V}_{s}{\rho }_{s}}}{\frac{{V}_{h}{\rho }_{h}}{{V}_{h}{\rho }_{h}+{V}_{s}{\rho }_{s}}+\frac{{V}_{s}{\rho }_{s}}{{V}_{h}{\rho }_{h}+{V}_{s}{\rho }_{s}}}$$

and

11 $${H}_{ci}=\frac{{\sigma }_{h}{H}_{h}\frac{{V}_{h}{\rho }_{h}}{{V}_{h}{\rho }_{h}+{V}_{s}{\rho }_{s}}+{\sigma }_{s}{H}_{s}\frac{{V}_{s}{\rho }_{s}}{{V}_{h}{\rho }_{h}+{V}_{s}{\rho }_{s}}}{{\sigma }_{h}\frac{{V}_{h}{\rho }_{h}}{{V}_{h}{\rho }_{h}+{V}_{s}{\rho }_{s}}+{\sigma }_{s}\frac{{V}_{s}{\rho }_{s}}{{V}_{h}{\rho }_{h}+{V}_{s}{\rho }_{s}}}.$$

The mass fraction of hard (*f*_h_^m^) and soft (*f*_s_^m^) magnetic phases are

12 $${f}_{h}^{m}=\frac{{V}_{h}{\rho }_{h}}{{V}_{h}{\rho }_{h}+{V}_{s}{\rho }_{s}}\,{\rm{and}}\,{f}_{s}^{m}=\frac{{V}_{s}{\rho }_{s}}{{V}_{h}{\rho }_{h}+{V}_{s}{\rho }_{s}},$$

respectively, where *V*_h_ *ρ*_h_ is the mass of hard phase and *V*_s_ *ρ*_s_ is the mass of soft phase. Accordingly, Eqs (10) and (11) become

13 $$\sigma ={\sigma }_{h}\,{f}_{h}^{m}+{\sigma }_{s}(1-{f}_{h}^{m})$$

and

14 $${H}_{ci}=\frac{{\sigma }_{h}{H}_{h}\,{f}_{h}^{m}+{\sigma }_{s}{H}_{s}(1-{f}_{h}^{m})}{{\sigma }_{h}\,{f}_{h}^{m}+{\sigma }_{s}(1-{f}_{h}^{m})},$$

respectively.

Eqs (13) and (14) can now be used to estimate the *σ*_s_ and *H*_ci_ of a two-phase magnet by only considering the mass fraction (*f*_h_^m^ or *f*_s_^m^) of hard and soft phases if their saturation magnetization and intrinsic coercivity are known.

## Experimental Validation

In order to validate the efficacy of Eqs (13) and (14), we synthesized four different composites, Sm_2_Co_7_/Fe-Co, MnAl/Fe-Co, MnBi/Fe-Co, and BaM/Fe-Co, by mixing hard and soft magnetic particles in an appropriate weight ratio and characterized them for magnetization and coercivity. It is noted that three different Fe-Co compositions, i.e., Fe_50_Co_50_, Fe_65_Co_35_, and Fe_80_Co_20_, were used for Sm_2_Co_7_/Fe-Co composites. The *σ*_s_ and *H*_ci_ of Fe_50_Co_50_, Fe_65_Co_35_, and Fe_80_Co_20_ are 236 emu/g and 75 Oe, 240 emu/g and 80 Oe, and 232 emu/g and 65 Oe, respectively.

**Figure 1:**
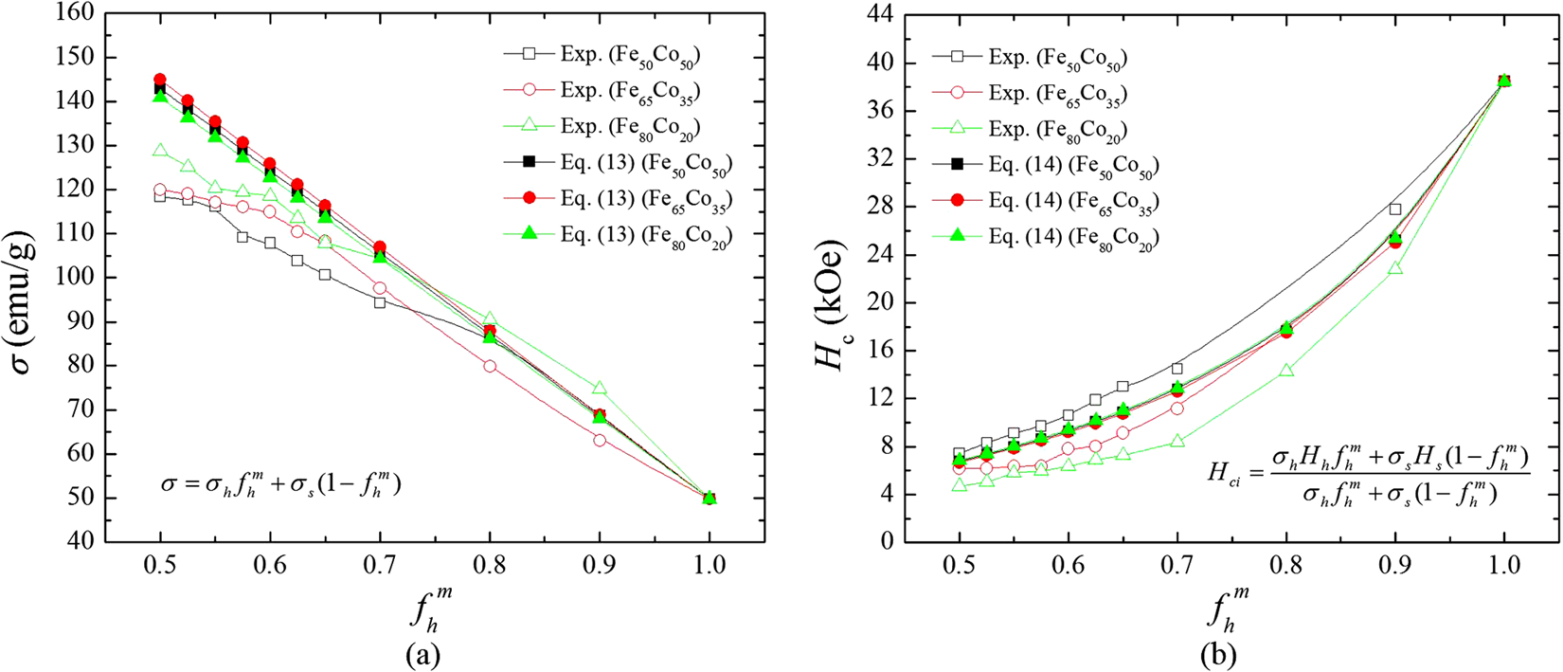
The mass fraction (*f*_h_^m^) of Sm_2_Co_7_ dependence of (**a**) saturation magnetization (*σ*_s_) and (**b**) intrinsic coercivity (*H*_ci_) of Sm_2_Co_7_/Fe-Co. The open and closed squares indicate experimental and calculated *σ*_s_ and *H*_ci_, respectively, for Sm_2_Co_7_/Fe_50_Co_50_ composites, circles indicate experimental and calculated *σ*_s_ and *H*_ci_ for Sm_2_Co_7_/Fe_65_Co_35_ composites, and triangles indicate experimental and calculated *σ*_s_ and *H*_ci_ for Sm_2_Co_7_/Fe_80_Co_20_ composites.

Figure 1(a) and (b) show the *f*_h_^m^ dependence of *σ*_s_ and *H*_ci_ for Sm_2_Co_7_/Fe-Co composite with various compositions of Fe-Co. The *σ*_s_ decreases linearly as the amount of hard phase (Sm_2_Co_7_) increases in Fig. 1(a). The experimental results (open symbol) are well fitted to our developed equation (13) (solid line). It is noted that at lower concentration of hard phase, deviation of experimental *σ*_s_ from the solid (theoretical) line is getting larger. In Fig. 1(b), experimental *H*_ci_ is excellently fitted to the developed equation (14), especially, for the composite with Fe_65_Co_35_.

**Figure 2:**
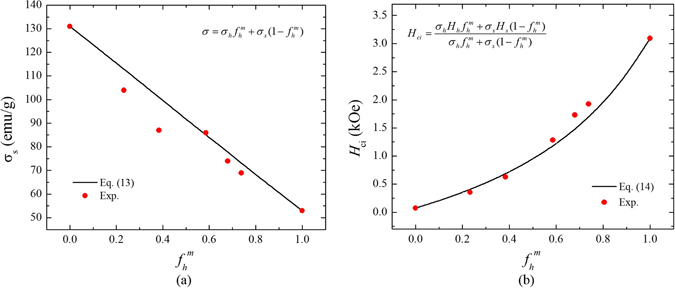
The mass fraction (*f*_h_^m^) of MnAl dependence of (**a**) saturation magnetization (*σ*_s_) and (**b**) intrinsic coercivity (*H*_ci_) of MnAl/Fe-Co. The black solid line and red closed circle indicate calculated and experimental data, respectively^8^.

As shown in Fig. 2(a) and (b), the *σ*_s_ of MnAl/Fe-Co composite linearly decreases by increasing the content of hard phase, and the *H*_ci_ increases by following the developed equation (14). Both experimental *σ*_s_ and *H*_ci_ are in good agreement with the present model.

**Figure 3:**
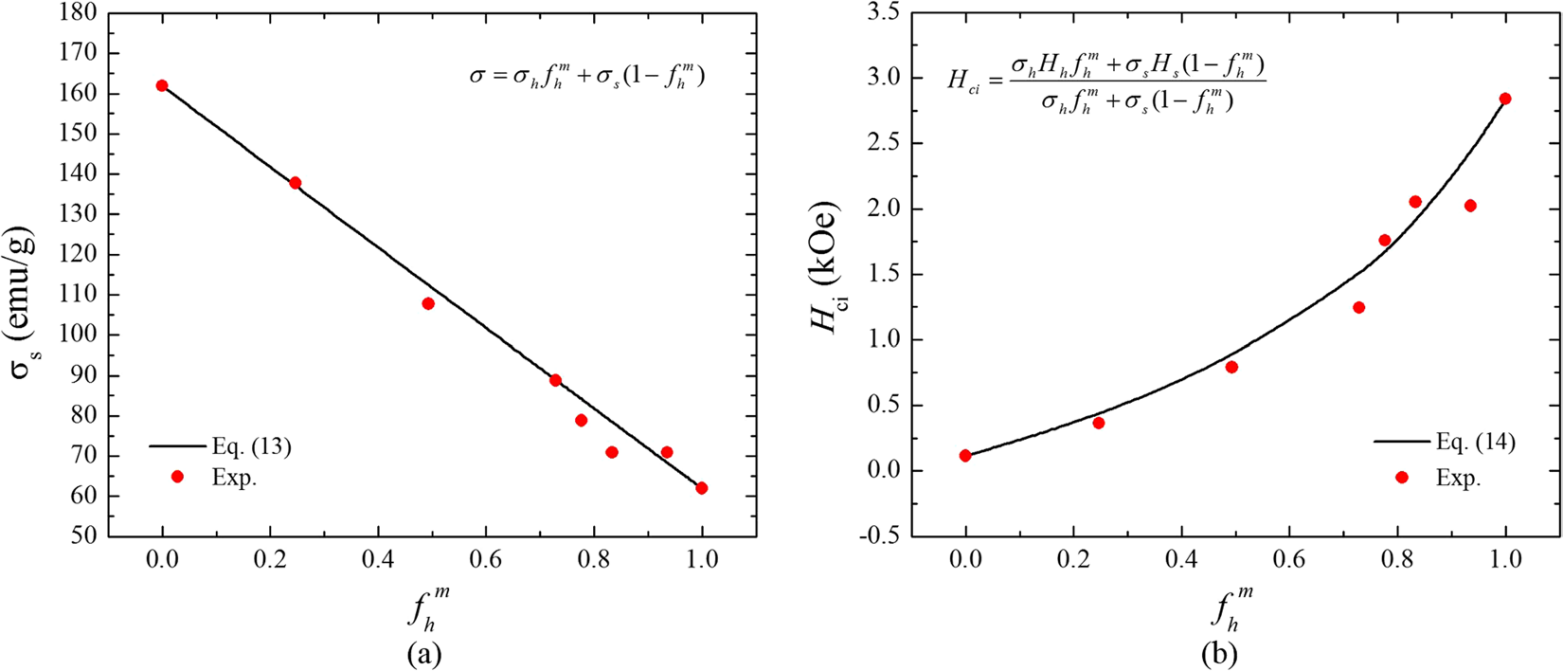
The mass fraction ( *f*_h_^m^) of MnBi dependence of saturation magnetization (*σ*_s_) and (**b**) intrinsic coercivity (*H*_ci_) of MnBi/Fe-Co. The black solid line and red closed circle indicate calculated and experimental data, respectively.

**Figure 4:**
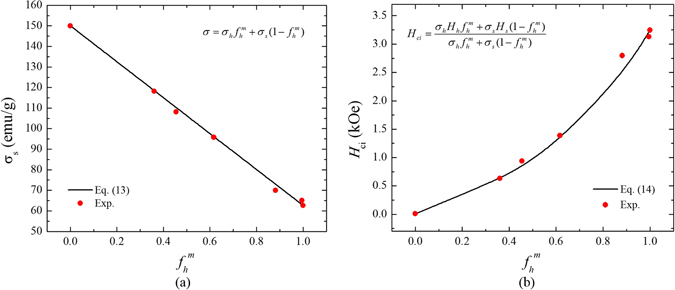
The mass fraction ( *f*_h_^m^) of BaM dependence of saturation magnetization (*σ*_s_) and (**b**) intrinsic coercivity (*H*_ci_) of BaM/Fe-Co. The black solid line and red closed circle indicate calculated and experimental data, respectively.

It was also found that the *σ*_s_ and *H*_ci_ of MnBi/Fe-Co composite magnet in Fig. 3 (a) and (b) are well fitted to Eqs (13) and (14). Lastly, Eqs (13) and (14) are also validated by the experimental *σ*_s_ and *H*_ci_ of BaM/Fe-Co composite shown in Fig. 4(a) and (b).

It is noted that a kink in the hysteresis loop becomes more obvious as the *f*_h_^m^ decreases, indicating weak or no exchange coupling (not shown in this paper). Therefore, regardless of exchange coupling, the present model can be used to estimate *σ*_s_ and *H*_ci_ of any powdered hard/soft magnet composite.

## Summary

In summary, we have modified the previously proposed models^1, 3^ for magnetization (*M*) and anisotropy constant (*K*) of a hard/soft composite magnet to use mass fraction instead of the volume fraction of hard or soft phase for magnetization (*σ*_s_) and intrinsic coercivity (*H*_ci_) of powdered hard/soft composite. Our modified equations have been validated by experimental *σ*_s_ and *H*_ci_ of Sm_2_Co_7_/Fe-Co, MnAl/Fe-Co, MnBi/Fe-Co, and BaM/Fe-Co composites. Regardless of exchange coupling, the developed equations can be used to predict the *σ*_s_ and *H*_ci_ of a powdered hard/soft composite magnet. The present model can provide guidance for the design of exchange coupled hard/soft composite magnets.

## References

[1] Park JH (2014). Electronic structure and maximum energy product of MnBi. Metals.

[2] Kneller EF, Hawig R (1991). The exchange-spring magnet: a new material principle for permanent magnets. IEEE Trans. Mag.

[3] Skomski R, Coey JMD (1993). Giant energy product in nanostructured two-phase magnets. Phy. Rev. B.

[4] Yu YS (2013). One-pot synthesis of urchin-like FePd–Fe_3_O_4_ and their conversion into exchange-coupled L1_0_–FePd–Fe nanocomposite magnets. Nano. Lett..

[5] Kittel C (1949). Physical theory of ferromagnetic domains. Rev. Mod. Phys..

[6] Stoner EC, Wohlfarth EP (1948). A mechanism of magnetic hysteresis in heterogeneous alloys. Phil. Trans..

[7] Neel L (1947). Proprietes d’un ferromagnetique cubique en grains fins. Comptes rendus (Paris).

[8] Park JH (2014). Magnetization and intrinsic coercivity for τ-phase Mn_54_Al_46_/α-phase Fe_65_Co_35_ composite. J. Magn..

